# Prevalence, associated factors, and clinical outcomes of *Helicobacter pylori* infection in pediatric populations in a war-torn urban environment in Eastern Democratic Republic of Congo: a mixed methods study

**DOI:** 10.1186/s12887-025-05588-7

**Published:** 2025-03-31

**Authors:** Emmanuel Busha Tibasima, Patrick Kumbowi Kumbakulu, Lundula Penge Chirac, Omari Ramazani, Tsumbu Byaruhanga Patrick, Kazembe Kamalo Olga, Gabriel Kakuru Shamavu, Mitangala Ndeba Prudence, Banga Mseza

**Affiliations:** 1 Department of Paediatrics and Child Health, Faculty of Clinical Medicine and Dentistry, Kampala International University, Ishaka-Bushenyi, Uganda; 2 Department of Paediatrics and Child Health, Université Libre Des Pays Des Grands Lacs, Goma, Democratic Republic of Congo; 3 Department Of Pediatric and Child Health Samaritan Doctor’S Pediatric Centre, Goma, Democratic Republic of Congo; 4 Departement of Epidemiology, Université Officielle de Ruwenzori, Butembo, Democratic Republic of Congo

**Keywords:** *Helicobacter pylori*, Prevalence, Associated factors, Gastrointestinal symptoms treatment outcomes, Pediatric populations, Eastern Democratic republic of Congo

## Abstract

**Background:**

*Helicobacter pylori (H. pylori)* infection remains a significant public health concern in developing countries, especially among pediatric populations where data are limited. In war-torn regions like the Eastern Democratic Republic of Congo, the impact of *H. pylori* infection on children's health may be exacerbated due to disrupted healthcare systems and limited resources.

**Methods:**

This mixed-methods study, incorporating both cross-sectional and prospective cohort designs, was conducted at Samaritan Doctor's Pediatric Centre between January 2020 and December 2022. The study enrolled 323 children aged 0 to 15 years presenting with gastrointestinal symptoms. Sociodemographic and clinical characteristics were assessed via questionnaire, and *H. pylori* stool antigen rapid tests were performed. Multivariate regression analyses were conducted. Participants were followed up and outcomes recorded after 30 days.

**Results:**

Of the 323 participants, 119 (36.80%) tested positive for *H. pylori* infection. Independent factors associated with *H. pylori* infection included age between 37 and 59 months (aOR: 9.76, 95% CI: 2.62–36.40, p = 0.001), caretaker’s occupation (aOR: 2.58, 95% CI: 1.19–5.54, p = 0.016), presence of pets at home (aOR: 0.371, 95% CI: 0.18–0.74, p = 0.005), drinking unsafe water (aOR: 0.13, 95% CI: 0.04–0.42, p = 0.001), anemia (aOR: 4.80, 95% CI: 1.12–20.53, p = 0.034), and presence of red blood cells in stool (aOR: 30.84, 95% CI: 13.97–68.10, p < 0.0001). Thirty days post-initial treatment with first-line medications (omeprazole, clarithromycin, and amoxicillin), 19.30% of patients remained positive for *H. pylori*.

**Conclusion:**

Children with occult blood in stool and microcytic anemia should be tested for *H. pylori* using stool antigen tests. Treatment with clarithromycin should be guided by local antibiotic resistance data. Hygiene education, including safe water practices and managing pet contact, is crucial due to their association with *H. pylori* infection.

## Background

### Introduction

*Helicobacter pylori (H. pylori)* is a microaerophilic, spiral-shaped, Gram-negative bacterium that infects the stomach lining. It is commonly associated with various gastrointestinal issues, presenting symptoms such as abdominal pain, nausea, and bloating [1]. This bacterium causes chronic inflammation of the stomach mucosa, leading to peptic ulcers and, if left untreated, more severe consequences like chronic active gastritis and gastric adenocarcinoma. Although *H. pylori* -related gastric cancer is rare in children, the infection itself remains clinically significant in the pediatric population due to its association with other gastrointestinal disorders. Globally, *H. pylori* infects approximately 50% of the population, making it one of the most widespread infections [2, 3].

While the prevalence of this infection has decreased in developed countries over the twenty-first century, *H. pylori* remains a significant health concern in low-income countries, often contributing to disability-adjusted life years (DALYs) [4]. *H. pylori* infection, although less likely to cause gastric cancer in children, still poses a significant health risk due to its potential to cause other severe gastrointestinal conditions [5]. In a review of data from population-based and clinical cancer registries, it was concluded that while gastric carcinoma is rare in children, a combination of *H. pylori* infection, tumour predisposition, and/or immunodeficiency appears to contribute significantly to the risk of developing gastric carcinoma at a young age [5]. Globally, approximately half of the population is affected by *H. pylori* infection, with prevalence rates ranging from 35 to 90% depending on factors like population diversity and geographic location [3]. The prevalence of this infection is not consistent across different societies; it varies based on geographic location, age, sex, and other socioeconomic factors, including ethnic group [6, 7]. In Japan, the prevalence of *H. pylori* infection among children ranged from 3.8% to 9.5%, whereas in Nigeria, it varied between 6.0% in the North-Central region and 28% in the South-Western region [8].

In African countries, where the prevalence of *H. pylori* generally exceeds 50%, it is crucial to understand the infection's impact on the pediatric population, especially in areas with limited healthcare resources. The highest prevalence rates have been reported in the Republic of the Congo (93.1%), the Democratic Republic of Congo (89.0%), Ethiopia (88.9%), Rwanda (75%), Cameroon (73.2%), and Burundi (70.8%)[8]. The association between *H. pylori* infection and gastric cancer is well established in adults. Evidence suggests that prolonged *H. pylori* infection, in conjunction with gastric atrophy and intestinal metaplasia, is associated with the development of intestinal-type and undifferentiated adenocarcinomas in adults[9].

In African nations, the incidence of gastric cancer is notably lower in pediatric populations compared to adults [5]. However, for children with *H. pylori*-associated gastric malignancies, there have been a few occurrences of gastric MALT lymphoma, but no cases of adenocarcinoma have been reported[10]. Nevertheless, regions with higher *H. pylori* prevalence demonstrate a more substantial disease burden, frequently exacerbated by insufficient healthcare infrastructure[5].

*H. pylori* infection in children is a significant public health concern influenced by a variety of factors. Age is a notable determinant, with older children frequently exhibiting higher infection rates[11, 12]. The educational level of the child's caretaker can also play a critical role, particularly in environments with varied levels of education and socioeconomic status[11, 13]. Environmental factors such as the presence of pets and consumption of unsafe water, along with familial elements like family size and bed-sharing practices, further contribute to the transmission within households [13, 14]. Abdominal symptoms including nausea, vomiting, and diarrhea, as well as associated complications like anemia characterized by hypochromia and macrocytosis, and gastrointestinal bleeding, as indicated by the presence of red blood cells in stool, are some clinical manifestations of *H. pylori* infection [9]. A family history of peptic ulcer disease (PUD) can indicate a genetic predisposition or shared environmental risk factors [10, 15]. Understanding these determinants is vital for developing targeted prevention strategies and improving diagnostic approaches for *H. pylori* infection in children.

The infection may occur in infancy and early childhood; if left untreated, the *H. pylori* infection generally persists throughout the individual's lifetime [3]. In comparison to adults, the understanding of *H. pylori* infection in children remains incomplete due to the prevalence of asymptomatic cases and limited research studies, which may contribute to an underestimation of the true prevalence [11]. However, childhood represents a critical period in the natural history of the infection [12]. Symptomatic children present with nonspecific symptoms such as abdominal pain, vomiting, or diarrhea[16]. Numerous studies concur on the interpersonal transmission of *H. pylori* infection within families as the primary route of contamination, with mother-to-child transmission being the most predominant mode. Oral-to-oral and oral-fecal transmission have been postulated through numerous papers as the principal routes of contamination[11]. In developing countries, *H. pylori* transmission is influenced by several factors, including low socioeconomic conditions, substandard drinking water, overcrowded living conditions, insufficient personal and environmental hygiene, and food contamination [17].

*H. pylori* treatment in children presents significant challenges, particularly in low- and middle-income countries[11]. The lack of routine culture and sensitivity testing in these regions complicates the selection of appropriate antibiotics, potentially leading to suboptimal treatment outcomes[16]. Healthcare providers in these regions often rely on empirical treatment strategies, which may inadvertently contribute to the spread of antibiotic resistance if ineffective regimens are used. This issue is further exacerbated by the rising global trend of clarithromycin resistance, which poses a threat to the efficacy of first-line treatment regimens [18]. Clarithromycin-based triple therapy has long been considered the standard first-line treatment for *H. pylori* infection in children. However, its effectiveness is increasingly questioned due to the growing prevalence of antibiotic resistance[16, 19]. Given these challenges, it is critical to evaluate the continued effectiveness of clarithromycin-based first-line treatments in pediatric populations, especially in areas where resistance patterns are poorly understood. The absence of culture and sensitivity testing in resource-limited settings creates a significant barrier to tailoring treatment approaches.

Over the past two decades, the eastern region of the Democratic Republic of Congo (DRC) has experienced armed conflicts, resulting in substantial population displacement toward more secure urban centres such as Goma city. Consequently, numerous families reside in overcrowded conditions, with suboptimal personal and environmental hygiene[20]. In Eastern DRC, data regarding the prevalence of *H. pylori* infection is limited, with research specifically focusing on children being particularly scarce. In this region, few studies have been conducted exclusively among adults or the general population in Bukavu, in the province of South-Kivu, and none among children [13, 14]. In conflict-affected areas, the impact of *H. pylori* infection on children's health may be exacerbated due to compromised healthcare systems and limited resources. However, specific data on *H. pylori* infection in children in war-affected regions are insufficient, necessitating further research to elucidate its prevalence, associated factors, and management outcomes in such contexts. To the best of our knowledge, there are no published data regarding *H. pylori* infection in children in North Kivu, particularly in Goma city. The primary objective of this research is to determine the prevalence, identify the associated factors, and investigate the treatment outcomes of *H. pylori* infection among children attending Samaritan Doctor's Pediatric Centre in Goma, Eastern DRC.

### Methods

#### Study design and setting

A mixed study design was conducted at Samaritan Doctor’s pediatric centre between January 2020 and December 2022. Initially, a cross-sectional study design was used to assess the prevalence of *H. pylori* infection and its associated factors among study participants. Subsequently, a prospective cohort study was carried out to follow patients who tested positive for *H. pylori*. The Samaritan Doctor’s pediatric centre is located in Goma city, North Kivu province, in Eastern DRC. This clinic run from Monday to Monday, (24 h) and serves an average of 15–20 patients daily.

#### Study population

All study participant aged between 0 and 15 years with at least one of the following nonspecific abdominal symptoms: abdominal pain, nausea, vomiting, diarrhea, bloating, and burping, were enrolled. Children who had been treated with any combinations of medicines used to treat of *H. pylori* infection including metronidazole, clarithromycin amoxicillin, bismuth subsalicylate, omeprazole or any other proton pump inhibitor in less than two weeks prior to the clinic visit were not enrolled in the study. Children with any cognitive impairments secondary to neurologic problems such as cerebral palsy or any other causes, were also excluded from the study because they couldn’t effectively describe abdominal symptoms.

#### Sample size determination and sampling technique

The sample size was calculated using Daniel’s formula: $n=\frac{{Z}^{2}* P*(1-P)}{{d}^{2}}$. Using findings from a study in Mbarara, in the Western Region of Uganda, where the proportion of *H. pylori* infection was 24.3% [17], P = 0.243. For 95% level of confidence, Z = 1.96 and d = 0.05. On substation, n = 283. After adding 14% to cater for loss of follow up, the required sample size was 323.

A total of 387 children with gastrointestinal symptoms and attending Samaritan Doctor’s pediatric centre were tested for enrolment into the study. Of these children, 64 were excluded from the study. Amongst them; 12 because having neurologic disabilities and 27 children were excluded because of having used a proton pomp inhibiter (omeprazole or Pantoprazole) or any combination of medications employed for the treatment or eradication of *H. pylori* (metronidazole, clarithromycin amoxicillin and bismuth subsalicylate). 348 children meet the inclusion criteria, however, 25 were excluded because they were accompanied by their caretakers, rather than parents/ legals guardians and we were unable to joined their parents to seek for consent. Finally, 323 children who the parents/ legal guardian consented were enrolled the study.

#### Data collection method and procedures

Caretakers of all children provided informed consent. They received consent forms to review and had the opportunity to ask questions. An assent form was provided for all study participant aged between seven and fifteen years. Depending on their reading ability, the forms were either read to them or read together. Both the children and their caretakers had the chance to seek clarification, and any unclear information was addressed by the investigator or research assistants. Subsequently, parental permission was obtained from the attender to allow the child’s participation in this research.

Privacy of participants was ensured during data collection. The interview was conducted in a separate room with limited access to their personal information to ensure participants’ privacy. In addition, no names were used on data collection tools, and data records were password-protected. Completed questionnaires were kept under lock and key and were accessible only by the principal investigator.

The investigators administered questionnaires in either French or the local languages (Swahili or Lingala) with the assistance of parents/caretakers for each child. Demographic information, including the child’s age, sex, residential address, parental occupation, access to drinking water, and exposure to various animals (such as dogs, cats, parrots, pigs, goats, ducks, and chickens) were collected.

**Fig. 1:**
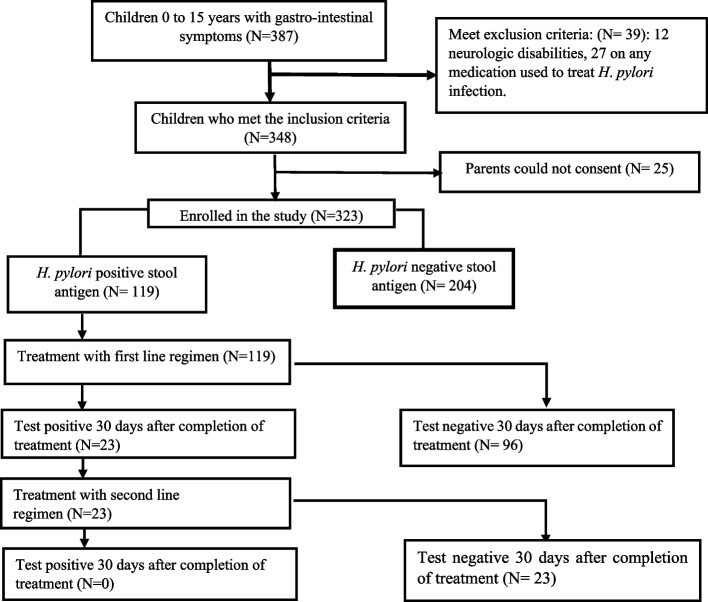
Study procedure

**Fig. 2:**
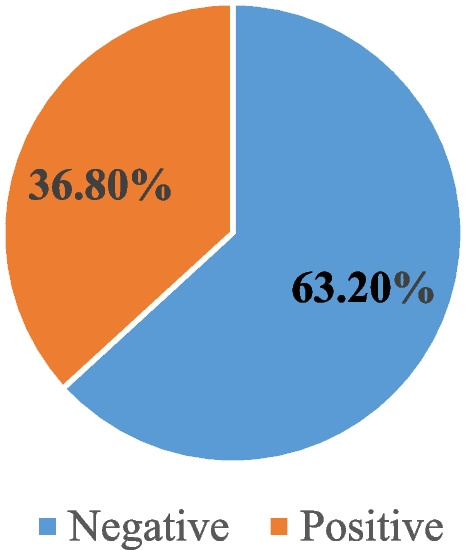
*Helicobacter pylori* antigen detection test from stool amongst children included in the study

The medical assessment included evaluating the presence of any of the following symptoms: abdominal pain, nausea, vomiting, diarrhea, bloating, burping, and weight loss. Additionally, the study considered the presence of family members or other individuals living with the child who had a history of peptic ulcer disease. Regarding clinical signs, the investigators assessed the following signs: pallor, jaundice, hepatomegaly, splenomegaly, and epigastric tenderness. Anthropometric measurements (weight and height) were also recorded during the interview (Figs. 1 and 2).

#### *H. pylori* diagnostic test

*H. pylori* antigens detection in a sample of fresh stools was carried out for infection diagnosis. Stool samples were obtained either immediately after clinical assessment or later in the day for participant who were not able to provide fecal specimen immediately. The sample was analyzed immediately after collection using *H pylori* Ag Rapid Test Cassette (feces), manufactured in China, which have a relative sensitivity of 98.8% and a relative specificity of 100%. If not tested within 6 h, the specimen collected was stored for 3 days at a temperature between 2–8 °C. A stool sample of approximately 1 to 2 ml (1–2 g) was collected in a clean, dry specimen collection container to maximize antigen detection.

#### Process fecal specimens

The procedure was carried out in accordance with the test manufacturer's instructions. For solid specimens, the cap of collection tube was unscrewed, and then the stool collection applicator was stabbed randomly into the fecal specimen in 3 different sites to collect approximately 50 mg of feces. For liquid specimens, following aspiration of the specimens, 2 drops were transferred into the sample tube containing the dilution buffer. After securely tightening the cap onto the sample tube, the specimen was vigorously shaken to thoroughly mix the sample with the dilution buffer. The tube was left to stand for 2 min and 2 drops of the sample solution were squeezed in the sample well of the cassette; the test was read after 10 min. The test device was discarded after interpreting the result to avoid any confusion. A result was considered positive in the presence of two lines, even if the test line exhibited less intensity than the control line. A result was considered negative when only the control line appears solely.

Another part of the stool specimen was used to look for evidence of red blood cell using Immunochemical Fecal Occult Blood Test (iFOBT). In Nigeria, when assessing patients with peptic ulcer and gastric disturbances, 71.4% of patients who tested positive for either *H. pylori* infection or fecal occult blood test were positive for both tests [21]. While false negatives may occur with FOBT alone, the combination of FOBT and *H. pylori* stool antigen tests enhances detection rates for upper gastrointestinal lesions. This approach provides a more comprehensive diagnostic tool, improving clinical management of *H. pylori*-related conditions and their complications.

Two ml of whole blood were also sampled from a peripheral vein of each study participant and immediately put into Ethylenediaminetetraacetic acid (EDTA) containing tube. This sample was used to evaluated the level of anemia amongst study participants. Full blood count was used to assess the level of anemia by providing valuable information on the levels of red blood cells, haemoglobin, haematocrit, mean corpuscular volume, mean corpuscular Haemoglobin and red cell distribution width and Mentzer index. Laboratory quality was observed by having inbuilt positive controls, double checking with previously established positive controls. Furthermore, two technicians independently performed the analysis they reached a consensus regarding the conclusive outcomes for each sample following discussions about challenging-to-interpret test results from the specimens.

#### Treatment

All patients diagnosed as positive for *H. pylori* were treated with a 14-day regimen. Follow-up testing was performed 4 weeks after completing the treatment using a non-invasive stool antigen assay. Patients who tested positive for *H. pylori* by fecal antigen testing were offered standard triple therapy, which included omeprazole (2 mg/kg twice a day), clarithromycin (15 mg/kg twice a day), and amoxicillin (50 mg twice a day).

The standard triple therapy was selected based on clinical guidelines for pediatric *H. pylori* eradication. Omeprazole, a proton pump inhibitor, reduces gastric acid secretion, creating a more favourable environment for the antibiotics to work effectively. Clarithromycin, a macrolide antibiotic, inhibits bacterial protein synthesis, while amoxicillin, a beta-lactam antibiotic, disrupts bacterial cell wall synthesis. This combination targets the bacteria directly and enhances the likelihood of successful eradication.

Thirty days post-treatment, all participants completed a post-treatment survey, and an *H. pylori* stool antigen test was repeated.

Participants who remained positive after initial therapy received an additional 14 days of quadruple therapy, consisting of bismuth subsalicylate (262 mg for children under 10 years and 524 mg for children over 10 years) four times a day, metronidazole (30 mg/kg four times a day), amoxicillin (50 mg/kg twice a day), and omeprazole (2 mg/kg twice a day). The quadruple therapy was chosen as second-line treatment due to its ability to address potential antibiotic resistance and provide a broader spectrum of antibacterial activity. Bismuth subsalicylate provides additional antimicrobial effects and helps protect the stomach lining, metronidazole is effective against anaerobic bacteria, and the combination of amoxicillin and omeprazole continues to target and suppress the *H. pylori* bacteria.

#### Follow-up of study participant

All children receiving *H. pylori* treatment were monitored for side effects and adherence. The follow-up was conducted through phone calls made every 5 days throughout the treatment course. This approach allowed healthcare providers to maintain regular contact with patients and their caregivers, enabling timely identification of any adverse reactions or compliance issues. During these calls, healthcare professionals inquired about the child's well-being, any experienced side effects, (nausea, abdominal discomfort, diarrhea, taste disturbances.) and adherence to the prescribed medication regimen. This proactive monitoring strategy facilitated early intervention, when necessary, potentially improving treatment outcomes and patient safety. Additionally, the frequent phone contact provided an opportunity to address any concerns or questions from parents or guardians, thereby enhancing overall treatment support and potentially increasing adherence rates.

#### Data management and analysis

Before concluding the interviews, all questionnaires were thoroughly reviewed to ensure they were fully completed. After the data was collected, no new information was added to the surveys. IBM SPSS 27.0 statistics for Windows (Armonk, NY: IBM Corp.) was used to organize, summarize, and analyse the data retrieved from completed questionnaires. *H. pylori* infection prevalence was calculated as proportion of patient with positive stool test among all participant enrolled in the study (from January 2020 to December 2022). Binary logistic regression was used to assess the factors associated with *H. pylori* infection both at bivariable and multivariable level. The crude and adjusted odds ratios (OR) with accompanying 95% confidence intervals (CI) were reported. A p-value of 0.05 or lower was regarded as statistically significant.

#### Respect of individual persons

The study participants’ legal guardians had the unrestricted right to withdraw their children from the study at any time, without the obligation to justify their decision. Importantly, the withdrawal from the study was not affecting the right of their children to receive medical care.

#### Benefits and risks

All investigations were free of charge aimed to improve patients care. The findings were shared with parents or caregivers. Participants who were tested positive received appropriate treatment. There were no major risks except some pain during phlebotomy or mild discomfort when moving the patient for physical examination or anthropometric measurement. Strict aseptic techniques were observed, and no infection occurred.

#### Justice

All eligible patients were given the opportunity to participate in the study regardless of their religious, racial, social or financial background. There was no any form of discrimination or preferential treatment.

## Results

### Baseline characteristic of study participants

#### Sociodemographic characteristic of study participants

Sociodemographic characteristics of study participants are presented in Table 1. The majority of children were aged between 5 and 15 years, 55.10% were female and most of caregivers had a level of education beyond the primary school.

**Table 1:** Baseline sociodemographic characteristic of study participants

Factors	Number	Percentage
**Age of children**		
0 to 12 months	18	5.60
13 to 24 months	11	3.40
25 to months	26	8.00
37 to 59 months	29	9.00
5 to 15 years	239	74.00
**Sex of children**		
Female	178	55.10
Male	145	44.90
**Occupation of parents**		
Formal	223	69.00
No formal	100	31.00
**Parent’s level of education**		
At most primary	30	9.30
More than primary	293	90.70
**Caretaker’s level of education**		
At most primary	115	35.60
More than primary	208	64.40
**Family size**		
< 5	42	13.00
> 5	281	87.00
**Bed sharing**		
Yes	288	89.20
No	35	10.80

##### Clinical and laboratory characteristics of study participants

Baseline clinical and laboratory characteristics of study participants are summarized in the Table 2. Abdominal pain was the major presenting complain of children. Almost half of study participant has red blood cell in the stool and 89.50% had a safe source of drinking water.

**Table 2:** Baseline clinical and laboratory characteristic of study participants

Factors	Number	Percentage
**Presence of Pets at home**		
Yes	140	43.30
No	183	56.70
**Source of drinking water**		
Safe	289	89.50
Unsafe	34	10.50
**Family history of PUD**		
Yes	122	37.80
No	201	62.20
**Abdominal pain**		
Yes	262	81.10
No	61	18.90
**Vomiting**		
Yes	86	26.60
No	237	73.40
**Diarrhea**		
Yes	26	8.00
No	297	92.00
**Nausea**		
Yes	127	39.30
No	196	60.70
**Weight loss**		
Yes	145	44.90
No	178	55.10
**Anemia**		
Yes	21	6.50
No	302	93.50
**Microcytosis**		
Yes	61	18.90
No	262	81.10
**Hypochromia**		
Yes	75	23.20
No	248	76.80
**RBC in stool**		
Yes	154	47.70
No	169	52.30

*RBC* red blood cell, *PUD* peptic ulcer disease

##### Prevalence of *H. pylori*

Of the 323 participants enrolled in the study, 119 (36.80%) was screened positive for H. pylori infection.

##### Treatment

Tables 3 and 4 are related to the treatment of H. pylori infection among study participant.

**Table 3:** First line therapy regimen with amoxicillin + clarithromycin + Omeprazole

Follow-up after 30 days	Regimen (amoxicillin + clarithromycin + Omeprazole)	
	**Number**	**Percentage**
Positive	23	19.30
Negative	96	80.70

**Table 4:** Second line therapy Regimen with bismuth subsalicylate + metronidazole + amoxicillin + omeprazole

Follow-up after 30 days	Regimen (bismuth + metro + amoxicillin + omeprazole)	
	**Number**	**Percentage**
Positive	0	0
Negative	23	100

The Table 3 present a follow-up conducted 30 days post-treatment. Out of the 119 children who were administered the first-line regimen—comprising of Amoxicillin, Clarithromycin, and Omeprazole—approximately one-fifth of study participant were tested positive for the infection 30 days after completing the treatment.

Table 4, as shown below, illustrates the second-line therapy regimen. This regimen, which includes Bismuth Subsalicylate, Metronidazole, Amoxicillin, and Omeprazole, was administered to patients who did not respond to the first-line treatment. Thirty days after completion of the treatment, all were tested negatives.

### Follow-up of study participant

The treatment was generally well-tolerated by the majority of patients. Common side effects reported included taste disturbances in 7/119 (5.90%) patients, nausea in 13/119 (10.90%), and mild diarrhea in 8/119 (6.70%). The adherence rate was high, with 110/119 (92.40%) patients following the treatment regimen as prescribed. Only a small proportion, 9/119 (7.60%), required additional support to complete the course.

### Factors associated with H. pylori infection amongst study participants

#### Bivariate analysis of Factors associated with H. pylori infection

The Table 5 below show the bivariate analysis of factors associated with *H. pylori* infection amongst study participants. The following factors were associated with *H. pylori* infections at bivariate binary logistic regression analysis: the age between 37 and 59 months (cOR 3.01, 95% C.I: 1.30–6.92,*p* = 0.01), Parent occupations (cOR 2.35, 95% CI:1.38–3.99, *p* = 0.002), parent’s level of education (At most primary: cOR 2.45 95% CI: (1.14–5.24, *p* = 0.02) presence of pet at the compound (cOR 2.01, 95% CI: 1.48–2.74, *p* = 0.001), positive family history of PUD (cOR 1.76, 95% CI: 1.10–2.79, *p* = 0.02), drinking unsafe water (cOR 4.9, 95% CI: 2.95–8.15, *p* = 0.001), weight loss (cOR 0.59, 95% CI: 0.37–0.95,* p* = 0.03) anemia (cOR 3.75, 95% CI:1.46 −9.58, *p* = 0.006) and presence of RBC in the stool ( cOR 26.5, 95% CI: 13.68 −51.3,* p* = < 0.0001).

**Table 5:** Bivariate analysis of Factors associated with H pylori infection

Variable	*H. pylori* ( +)		*H. pylori* (-)		Bivariate analysis	
	**N°**	**%**	**N°**	**%**	**P value**	**cOR(95%CI)**
**Age**						
0—12 months	7	38.90	11	61.10		1
13—24 months	2	18.20	9	81.80	0.72	1.2 (0.44–3.20)
25—36 months	16	61.50	10	38.50	0.27	0.42 (0.088–1.97)
37—59 months	11	37.90	18	62.10	0.01	3.01 (1.30–6.92)*
5—15 years	83	34.70	156	65.30	0.73	1.15(0.51–2.54)
**Sex**						
Female	67	37.60	111	62.40		1
Male	52	35.90	93	64.10	0.74	1.08(0.68–1.70)
**Caretaker’s level of education**						
At most primary	48	41.70	67	58.30	0.18	1.38 (0.86–2.20)
More than primary	71	34.10	137	65.90		1
**Occupation of parents**						
Formal	95	42.60	128	57.40		1
No formal	24	24	76	76	0.002	2.35 (1.38–3.99)*
**Parent’s level of education**						
At most primary	17	56.70	13	43.30	0.02	2.45 (1.14–5.24)*
More than primary	102	34.80	191	65.20		1
**Family size**						
< 5	12	28.60	30	71.40		1
> 5	107	38.10	174	61.90	0.24	1.54 (0.97–2.45)
**EBF**						
Yes	86	35.50	156	64.50		1
No	33	40.70	48	59.30	0.4	0.80 (0.47–1.34)
**Pets**						
Yes	39	27.90	101	72.10	0.001	2.01(1.48–2.74)*
No	80	43.70	103	56.30		1
**Source of drinking water**						
Safe	95	32.90	194	67.10		1
Unsafe	24	70.60	10	29.40	0.001	4.9 (2.95–8.15)*
**Bed sharing**						
Yes	110	38.20	178	61.80	0.15	1.79 (0.80 −3.95)
No	9	25.70	26	74.30		1
**Family history of PUD**						
Yes	55	45.10	67	54.90	0.02	1.76 (1.10–2.79)
No	64	31.80	137	68.20		1
**Weight loss**						
Yes	44	30.30	101	69.70	0.03	0.59 (0.37–0.95)*
No	75	42.10	103	57.90		1
**Anemia**						
Yes	14	66.70	7	33.30	0.006	3.75 (1.46 −9.58)*
No	105	34.80	197	65.2		1
**Microcytosis**						
Yes	28	45.90	33	54.10	0.10	1.59 (0.90–2.80)
No	91	34.70	171	65.30		1
**Hypochromia**						
Yes	32	42.70	43	57.30	0.23	1.37 (0.81–2.33)
No	87	35.10	161	64.90		1
**RBC in stool**						
Yes	106	68.80	48	31.20	< 0.0001	26.5 (13.68 −51.3)*
No	13	7.70	156	92.30		1

*cOR* crude odds ratio, *CI* confidence interval, *RBC* red blood cell, *PUD* peptic ulcer disease, *EBF* exclusive breastfeeding

^*^Statistically significant (*p* < 0.05); + tested positive;—tested negative

#### Multivariate analysis of factors associated with *H. pylori* infection

The Table 6 below show the multivariate analysis of factors associated with *H. pylori* infection amongst study participants. The following factors were significantly and independently associated with *H. pylori* infections: the age between 37 and 59 months (aOR 9.76, 95% C.I: 2.62–36.40, *p* = 0.001), parent’s occupations (aOR 2.58, 95% C.I:1.19–5.54, *p* = 0.016), presence of pet at the compound (aOR = 2.43, 95% CI: 1.17–5.06; *p* = 0.02); drinking unsafe water (aOR = 8.7, 95% CI: 2.49–30.41, *p* = 0.001) anemia (aOR 4.804, 95% C.I: 1.12–20.53 *p* = 0.034) and presence of red blood cells in the stool (aOR 30.84, 95% C.I:13,97–68,10,* p* = < 0.0001).The odds of *H. pylori* infection were 30.84 times higher among children with red blood cells in the stool compared to those who didn’t. The odds *H. pylori* infection was higher by 9.76 amongst children aged between 37 and 59 moths compare to others age group. Anemia increases the odd of *H. pylori* infection by 4.8. Others characteristics is showed in the Table 6 below.

**Table 6:** Multivariate analysis of Factors associated with H. pylori infection

Variable	Bivariate analysis		Multivariate analysis	
	***p*- value**	**cOR (95%CI)**	**p-value**	**aOR (95%CI)**
**Age**				
0—12 months		1		
13—24 months	0.72	1.2 (0.44–3.20)	0.59	1.49 (0.354–6.25)
25—36 months	0.27	0.42 (0.088–1.97)	0.48	2,16 (0.261–17.99)
37—59 months	0.01	3.01 (1.30–6.92)	0.001	9.76 (2.621–36.40)*
5—15 years	0.73	1.15(0.51–2.54)	0.30	1.792 (0.59—5.43)
**Occupation of parents**				
Formal		1		
No formal	0.002	2.35 (1.38–3.99)	0.016	2.58 (1.19–5.54)*
**Level of education of parents**				
Not more than primary	0.02	2.45 (1.14–5.24)	0.36	1.678 (0.550–5.11)
More than primary		1		
**Presence of Pets at home**				
Yes	0.001	2.01(1.48–2.74)	0.02	2.43 (1.17–5.06)*
No		1		
**Source of drinking water**				
Safe		1		
Unsafe	0.001	4.9 (2.95–8.15)	0.001	8.7 (2.49–30.41)*
**Family history of PUD**				
Yes	0.02	1.76 (1.10–2.79)	0.062	1.940 (0.97–3.891)
No		1		
**Weight loss**				
Yes	0.03	0.59 (0.37–0.95)	0.366	0.735 (0.378–1,432)
No		1		
**Anemia**				
Yes	0.006	3.75 (1.46 −9.58)	0.034	4.804 (1.12–20.53)*
No		1		
**RBC in stool**				
Yes	< 0.0001	26.5 (13.68–51.3)	< 0.0001	30.84(13,97–68,10)*
No		1		

*aOR* Adjusted odds ratio, *cOR* Crude odds ratio, *CI* Confidence interval

^*^Statistically significant (*p* < 0.05; + tested positive;—tested negative; RBC = red blood cell; PUD = peptic ulcer disease

## Discussion

### Prevalence of H. pylori infection amongst children aged 0 to 15 years

This study conducted a longitudinal investigation of *H. pylori* infection in a cohort of children aged 0 to 15 years in Goma, North Kivu province, Eastern Democratic Republic of Congo (DRC). The findings revealed a prevalence of 36.80% among children attending the Samaritan Doctor's pediatric centre. This rate suggests that *H. pylori* infection is endemic among children in Goma City, a region affected by protracted conflict and population displacement. These results have significant implications for public health in the area and contribute to the understanding of the global distribution of this infection.

These results align with a recent systematic review suggesting that nearly half of the world’s population is infected with *H. pylori*, highlighting its global prevalence [22]. Our findings are consistence with some researches undertaken in surrounding countries. A comprehensive review and meta-analysis from 2011 to 2016 found that the global childhood prevalence of *H. pylori* infection was 33% [23]**.** In Kasese district, Uganda, the prevalence of *H. pylori* infection among children was 37.4% evaluated with the immunochromatographic rapid test (IRT) [24]. Similarly, a retrospective study conducted over five years in Kampala observed a prevalence of 35.7% in patients with gastrointestinal symptoms [25].

On the other hand, the prevalence observed in our study was lower than the 79.1% expected in African countries [22]**.** This prevalence is also lower compared to a study conducted on Ethiopian school children, which reported a *H. pylori* prevalence rate of 65.7% [26]. Higher prevalence rates were also reported in Ugandan children with sickle cell anemia (49%) [1] and in Cameroon (51.5%) [27]. Our study’s prevalence was higher compared to the prevalence reported in Mbarara, western Uganda (24.3%) [17] and in Havana, Cuba (5%) [28]. The prevalence of *H. pylori* infection varies from region to region worldwide, and among different population groups, study settings and type of diagnosis tests. Goma city, unlike Mbarara and Havana which reported low prevalence rates, is located in a war-torn area with massive population movement, overcrowding, and water supply problems. Thus, may partially explain the difference in the prevalence of *H. pylori* infection. The variation in prevalence rates across different studies highlights the importance of standardized diagnostic methods in epidemiological research. The use of serologic tests in some studies, which cannot distinguish between active and past infections, may lead to overestimation of prevalence rates. This emphasizes the need for more accurate, non-invasive diagnostic tools for large-scale screening, especially in resource-limited settings. The unique conditions in Goma, including prolonged conflict, overcrowding, and water supply issues, likely contribute to the observed prevalence. These findings underscore the need for a multifaceted approach to addressing *H. pylori* infection, encompassing improvements in sanitation, water quality, and living conditions, alongside medical interventions.

### Factors associated to *H. pylori* infection among study participant

In this study, several factors were found to be significantly and independently associated with *H. pylori* infection among children aged 0 to 15 years. These factors included age between 37 and 59 months, caretaker’s occupation, presence of a pet in the compound, consumption of unsafe drinking water, anemia, and the presence of red blood cells (RBC) in the stool.

Children aged 37–59 months were 9.76 times more likely to be infected, aligning with previous studies reporting *H. pylori* acquisition between the second and third year of life [29]. The findings are consistent with research from other developing countries, which have reported high infection rates in children under 5 years. For instance, studies in Gambia [30], Uganda [17], Vietnam, Mexico, Egypt, and Bangladesh, all demonstrated early acquisition of *H. pylori* infection, with prevalence rates ranging from 22.6% to 80% in children under 5 years. [15, 31–33]. The period below 5 years of age represents a critical window for *H. pylori* infection in childhood due to multiple factors. During this developmental stage, children are establishing hygiene habits and may experience increased exposure to infection sources [34]. Their immune systems are still in the process of maturation, rendering them more susceptible to colonization [11]. This period frequently coincides with entry into preschool or daycare environments, facilitating close contact with other children[13]. Environmental conditions such as high-density living areas and inadequate sanitation, in conjunction with dietary habits and exposure to contaminated food and water sources, contribute to the elevated prevalence [11, 35]. These findings emphasize the necessity for targeted public health interventions to mitigate *H. pylori* transmission in early childhood.

In this study, children with parents having informal occupations were 2.58 times more likely to be infected with *H. pylori* compared to those with parents in formal occupations. This finding aligns with recent research indicating that socioeconomic factors, including parental occupation, can influence *H. pylori* infection rates in children[36]. Informal occupations often correlate with lower income and less stable living conditions, which may contribute to increased exposure to *H. pylori* through contaminated water and poor sanitation[34, 37]. Further studies are needed to explore the mechanisms underlying this association and to develop targeted interventions to reduce *H. pylori* infection in vulnerable populations.

Children consuming unsafe water were 8.7 times more likely to have *H. pylori* compared to those who did not. This finding underscores the critical role of water quality in the transmission of *H. pylori*. Goma city and its surrounding areas face a severe drinking water shortage, exacerbated by persistent insecurity, massive population displacement, and a lack of investment in water infrastructure[20]. Numerous studies indicate that various types of water, including drinking water, wastewater, surface water, and groundwater, can be potential reservoirs for *H. pylori* infection[38, 39]. Unsafe drinking water can harbour the bacterium, leading to infection through ingestion, and children are infected through consumption of contaminated water[39]. The bacterium can survive in contaminated water sources, and upon consumption, it colonizes the stomach lining, causing infection[11]. Recent studies, have also demonstrated a strong association between drinking unsafe water and *H. pylori* infection [35, 40]. In Istanbul, Turkey, low socio-economic status, dinking unsafe water, and consumption of uncooked vegetable was reported as important source of this infection [15]. In Havana, Cuba, drinking potable water was protective against *H. pylori* infection [28]. This reinforces the necessity for targeted interventions to ensure access to safe drinking water, thereby reducing the risk of *H. pylori* infection and improving public health outcomes.

In our study, children who had pets at home were 2.43 times more likely to be infected with *H. pylori* compared to those who did not. The association between *H. pylori* infection in children and the presence of pets in the household is a subject of ongoing research and debate within the medical community. Some studies suggest that pet ownership, particularly of dogs, and cat may be associated with an increased risk of *H. pylori* infection in children[41]. Pets may serve as potential reservoirs or vectors for the bacteria, facilitating transmission through close contact or contaminated environments[42]. However, other studies have found no significant correlation between pet ownership and *H. pylori* infection rates in children. Machine learning algorithms were employed to identify risk factors for *H. pylori* infection among school children in Ethiopia. While several risk factors were identified, pet ownership was not found to be a significant predictor of *H. pylori* infection[35]. Similarly, various risk factors for *H. pylori* infection in children were examined but did not establish a significant correlation between pet ownership and infection rates[11]. These conflicting findings underscore the complexity of *H. pylori* transmission and the necessity for further investigation.

Children with red blood cells (RBCs) in their stool were 30.84 times more likely to be infected by *H. pylori* compared to those without RBCs. Fecal occult bleeding is well-documented in *H. pylori* infection. An increased risk of occult gastrointestinal bleeding in *H. pylori*-positive children was reported in 2018 [43]. In Nigeria, 36.67% of patients with non-specific abdominal symptoms tested positive for both *H. pylori* and fecal occult blood [21]. In India, 20.7% of the patients with upper gastrointestinal bleeding were *H. pylori* positive [44]. and in Egypt, 65.4% of such children were infected. [45]. Gastric mucosal inflammation often chronic-active or nodular gastritis—is common in *H. pylori*-infected children [46]. Antral gastritis and duodenal ulcers are recognized causes of fecal occult bleeding in childhood[10]. These findings highlight the importance of early detection and treatment of *H. pylori* infection in children, especially when blood is detected in stool samples.

Children with anemia were 4.8 times more likely to be infected by *H. pylori* compared to those without anemia. *H. pylori* infection can lead to anemia by impairing the absorption of essential micronutrients and elevating the production of hepcidin in liver cells[47]. Additionally, *H. pylori* cause chronic gastritis, which impairs iron absorption and can lead to gastric ulcers, resulting in chronic blood loss. The infection can also interfere with the absorption of vitamin B12, leading to megaloblastic anemia. Furthermore, *H. pylori* induces systemic inflammation, disrupting red blood cell production and contributing to anemia[48]. This finding aligns with Asiimwe et al. (2023), who reported a significant association between *H. pylori* infection and anemia in dyspeptic patients in Uganda[40]. In a population-based survey conducted in Alaska, the prevalence of anemia and iron deficiency among children with *H. pylori* infection was 17% and 15%, respectively [49]. A recent systematic review revealed that children with *H. pylori* infection were at a higher risk of developing anemia compared to non-infected children [47]. Additionally, a retrospective case–control study in Saudi Arabia assessed the association between *H. pylori* infection and anemia across all age groups, finding that the prevalence of anemia, iron deficiency anemia (IDA), and microcytic anemia was higher among the infected group compared to uninfected children [50]. These findings underscore the importance of considering *H. pylori* infection in pediatric patients with anemia.

### Treatment of H. *pylori* infection amongst children aged 0 to 15 years with H. pylori

In our study, 19.3% of patients treated with first-line drugs, including omeprazole, clarithromycin, and amoxicillin, remained positive for *H. pylori* 30 days after completing their initial treatment, indicating an eradication rate of 80.7%. In contrast, 100% of patients who did not respond to the first-line regimen were successfully treated with quadruple therapy consisting of bismuth subsalicylate, metronidazole, amoxicillin, and omeprazole.

This finding underscores the importance of considering alternative treatment strategies for patients who do not respond to initial therapy. Quadruple therapy has shown to be highly effective in such cases, providing a viable option for achieving complete eradication of *H. pylori.*

Our results align closely with a study carried out in Swedish children from 2005 to 2016, where 21% of cases showed resistance to clarithromycin[51]. This result is also consistent with a prospective, open, comparative, and cross-sectional study involving 228 Chinese children aged between 6 and 18 years, where the eradication rate was as low as 74.1% for standard triple therapy; however, in their study, the eradication rate with bismuth-based quadruple therapy was 89.9%[19]. In another study conducted in Portugal, the resistance rates were 6.7% for amoxicillin, 3.3% for metronidazole, and 23.3% for clarithromycin [52]. The eradication rates following a standard triple therapy of 7 to 14 days fall below 90% if clarithromycin resistance rates exceed 5%[18].

The current recommendation of international consensuses on *H. pylori* eradication therapy suggests that only regimens that reliably produce eradication rates of more than 90% should be used for empirical treatment[18]. In areas where the eradication rate of *H. pylori* infection exceeds 90%, most guidelines recommend a treatment course lasting 7 to 14 days, consisting of the standard triple therapy as the first-line approach[18]. A culture and sensitivity test are recommended if there is either unknown clarithromycin resistance or if the resistance level is more than 5% before initiating patients on clarithromycin-based triple therapy regimens [52]. In our study area, the prevalence of clarithromycin resistance and other drugs used as first-line therapy is unknown; this may explain the low rate of eradication compared to other studies conducted worldwide. Additionally, we did not perform culture and antibiotic susceptibility testing, so this result may be explained by poor adherence to therapy and other uncontrolled factors.

These findings highlight the necessity for further research into the prevalence of antibiotic resistance in our study area and the importance of adherence to therapy. Future research should focus on conducting culture and sensitivity tests to tailor treatment plans more effectively and explore additional strategies to improve adherence to *H. pylori* treatment regimens. By addressing these challenges, we can enhance the overall effectiveness of *H. pylori* eradication therapies and reduce the incidence of treatment failure.

### Study limitations and strength

This study presents several limitations that may have influenced the observed prevalence. The focus on children in healthcare settings potentially introduces selection bias, as the sample may not be representative of the overall population of Goma City. Consequently, the prevalence observed in this study may not accurately reflect the true prevalence in the broader population.

The utilization of the stool antigen test, despite its high sensitivity and specificity, has inherent limitations. The inability to perform endoscopy and gastric biopsy, which are considered the gold standard for *H. pylori* infection diagnosis, represents a significant constraint. Furthermore, the absence of culture and susceptibility testing limits the capacity to detect antibiotic resistance.

The reliance on self-reported data from patients and caregivers regarding treatment adherence and side effects introduces potential reporting bias. The accuracy of such self-reported information can be influenced by factors such as recall bias and the willingness of participants to report accurately.

Despite these limitations, our study has significant strengths. To our knowledge, this is the first study conducted in eastern DRC, a war zone with a crowded population, to evaluate the rate of treatment failure after using clarithromycin-based therapy as the first line. Our study provides important baseline data on the prevalence of *H. pylori* and its resistance to antibiotics in this region, which can inform future research and treatment strategies.

## Conclusion

This was, at our knowledge at the time, the first study in eastern DRC, specifically Goma city. The prevalence of *H. pylori* amongst children aged 0 to 15 years attending Samaritan Doctors pediatric centre was low than expected in African countries. Factors independently associated to *H. pylori* infection, identified through a multivariate binary logistic regression, included the age of the child, caretaker’s occupations, presence of pet at the compound, drinking unsafe water, anemia and presence of red blood cells in the stool. The eradication rate of *H. pylori* infection using standard clarithromycin based triple therapy was lower than the recommended of most current guidelines. Children presenting with, occult blood in the stool and microcytic anemia should be systematically screened for *H. pylori* infection using stool antigen test, which provides evidence of an active infection.

The use of clarithromycin-based therapy as first line of treatment to eradicate *H. pylori* infection should be guided by local data on antibiotic resistance. Patients and caretakers should be counselled about hygienic measures, including drinking potable safe water, and risk of close contact with pet at home, which is a real risk factor for *H. pylori* infection.

## Data Availability

Data is available upon request to the primary investigator.

## Abbreviations

aOR: Adjusted odd ratio
CI: Confident interval
cOR: Crude odd ratio
DALYs: Disability Adjusted Life Years
DRC: Democratic Republic of Congo
EDTA: Ethylenediamine tetraacetic acid
GIT: Gastrointestinal tract
IDA: Iron deficiency anemia
iFOBT: Immunochemical Fecal Occult Blood Test
IRT: Immunochromatographic rapid test
RBC: Red Blood Cells
UN: United Nations
